# Heat-Killed *Lactobacillus plantarum* beLP1 Attenuates Dexamethasone-Induced Sarcopenia in Rats by Increasing AKT Phosphorylation

**DOI:** 10.3390/biomedicines13071668

**Published:** 2025-07-08

**Authors:** Jinsu Choi, Eunwoo Jeong, Harang Park, Hye-Yeong Song, Juyeong Moon, Min-ah Kim, Bon Seo Koo, Jin-Ho Lee, Jong Kwang Hong, Kwon-Il Han, Doyong Kim, Han Sung Kim, Tack-Joong Kim

**Affiliations:** 1 Division of Biological Science and Technology, Yonsei University, Wonju 26493, Republic of Korea; wlstnbnm@naver.com (J.C.); jew0108@naver.com (E.J.); phraa@naver.com (H.P.); shy9987@naver.com (H.-Y.S.); mjy@yonsei.ac.kr (J.M.); mina1218@yonsei.ac.kr (M.-a.K.); kbs1349@yonsei.ac.kr (B.S.K.); drlogos@naver.com (J.-H.L.); jongkwang.hong@yonsei.ac.kr (J.K.H.); 2 Research & Development Center, bereum Co., Ltd., Wonju 26362, Republic of Korea; kihan@bereum.com; 3 Department of Biomedical Engineering, Yonsei University, Wonju 26493, Republic of Korea; dykim2650@naver.com (D.K.); hanskim@yonsei.ac.kr (H.S.K.); 4 Research & Development Center, Doctor TJ Co., Ltd., Wonju 26494, Republic of Korea

**Keywords:** sarcopenia, *Lactobacillus plantarum*, postbiotic, beLP1, dexamethasone

## Abstract

**Background/Objectives**: Sarcopenia is an age-related disease resulting in muscle mass deterioration and declining strength and functional ability. Muscle protein degradation pathways are activated through the ubiquitin–proteasome system, which is integral to the pathogenesis of sarcopenia. This study examined the capability of *Lactobacillus plantarum* beLP1 as a postbiotic ingredient of kimchi that prevents sarcopenia. **Methods**: We evaluated cell viability and measured diameters in a C2C12 myotube damage model and muscle volume, muscle weight, muscle strength, and the expression of muscle degradation proteins MuRF1 and Atrogin-1 in dexamethasone-induced sarcopenic model rats using a heat-killed beLP1 strain. **Results**: beLP1 had no cytotoxic effects on C2C12 and prevented dexamethasone-induced cellular damage, suggesting its role in muscle protein degradation pathways. beLP1 treatment significantly prevented the dexamethasone-induced reduction in myotube diameter. In a dexamethasone-induced sarcopenic rat model, oral beLP1 significantly mitigated muscle mass decline and prevented grip strength reduction. Microcomputed tomography demonstrated that beLP1 reduced dexamethasone-induced muscle volume loss. beLP1 treatment reduced Atrogin-1 and Muscle RING-finger protein-1 (MuRF1) and the transcription factor Forkhead box O3 alpha (FoxO3α), which triggers muscle protein breakdown. beLP1 exerts protective effects by inhibiting the ubiquitin-proteasome system and regulating FoxO3α signaling. It increased AKT (Ser473) phosphorylation, which affected muscle protein synthesis, degradation, and cell survival, suggesting its potential to prevent sarcopenia. **Conclusions**: Heat-killed *Lactobacillus plantarum* beLP1 alleviates muscle mass wasting and weakness in a dexamethasone-induced sarcopenia model by regulating muscle protein degradation pathways and signaling molecules. Thus, postbiotics may be functional ingredients in sarcopenia prevention.

## 1. Introduction

Sarcopenia is the loss of muscle function, strength, and mass associated with aging. Consequently, patients with sarcopenia experience diminished physical performance, which ultimately leads to the loss of independence in daily living activities. Sarcopenia increases the rates of falls and fractures and drastically impairs the quality of life [1,2]. The aging global population is an increasingly important social issue [3,4]. The World Health Organization officially bestowed the disease status on sarcopenia with the International Classification of Diseases (ICD-10) code M63.84 in 2016 [5]. However, there is no approved pharmacological treatment for sarcopenia currently, but preventive management, such as exercise and nutrition management, is often prescribed [6,7,8].

Sarcopenia is directly associated with muscle and cellular changes in muscle cells during aging, particularly ubiquitin–proteasome system (UPS) activation, which is the primary response to muscle protein expression [9,10]. Within this pathway, muscle RING-finger protein-1 (MuRF1) and Atrogin-1 E3 ubiquitin ligases play key roles in enhancing protein degradation in the muscles [11,12]. These ligases enhance the ubiquitination of protein targets in the muscles, marking them for breakdown by the 26S proteasome [13]. Earlier studies have frequently reported that the expression of E3 ubiquitin ligases, especially MuRF1 and Atrogin-1, increases with aging, leading to the elevated breakdown of muscle proteins [14,15]. Therefore, these ligases destabilize protein homeostasis in the muscles, leading to skeletal muscle atrophy and reduced muscle strength and mass [16].

Aging reduces the number of satellite cells that recover and regrow muscles and decreases the levels of central anabolic hormones, such as the growth hormone and insulin-like growth factor-1, thus suppressing muscle repair progressively [17,18,19]. Oxidative stress associated with aging activates the formation of reactive oxygen species, which leads to mitochondrial injury and induces apoptosis in muscle cells, thereby promoting muscle weakness and loss [20,21].

In recent years, probiotics have attracted interest as potential next-generation therapeutic agents [22]. Probiotics are functional molecules with known beneficial activities, ranging from gut microbiota modulation to antioxidation and strengthening of the immune system [23]. However, probiotics consist of living bacteria and therefore carry the risk of causing pathogenic infection, especially in people with weakened immune systems [24].

To counter these issues, postbiotics have been established as safer alternatives to probiotics [25]. Postbiotics are composed of microbial metabolites and cell lysates of peptidoglycans that possess the physiological benefits of immune regulation, antioxidation, and ameliorating inflammation [26]. Compared to probiotics, postbiotics maintain their desired properties with increased safety. Studies have reported that heat-killed *Lactobacillus* strains and their constituents can inhibit protein breakdown in muscles and induce protein synthesis, which may be helpful in developing promising therapeutic approaches for the prevention and treatment of sarcopenia. However, research on the exact mechanisms and effects of postbiotics on muscle function remains in its nascent stages [27].

Dexamethasone is a synthetic steroid and glucocorticoid with anti-inflammatory and immunosuppressive effects [28]. However, it decreases muscle protein synthesis and inhibits protein degradation in the long term, causing sarcopenia. Because of these effects, dexamethasone is commonly used to induce sarcopenia in cells and animal models [29].

Dexamethasone-induced sarcopenia models may serve as viable alternatives to naturally aged models for investigating skeletal muscle loss [30] compared dexamethasone-treated young mice and naturally aged mice and reported comparable reductions in muscle mass, grip strength, muscle fiber size, and contractile function in both models. Moreover, muscle atrophy-related genes such as *MuRF1* and *Atrogin-1*, were upregulated in both models indicating similar molecular responses involved in muscle degradation. Although differences in mitochondrial DNA damage and certain gene expression profiles exist, the functional and histological similarities highlight the dexamethasone-induced model as a suitable and reproducible alternative for preclinical studies on sarcopenia.

Curcumin, a polyphenolic compound derived from Curcuma longa, ameliorates dexamethasone-induced muscle atrophy by reducing oxidative stress, suppressing the expression of atrophy-related genes such as MuRF1 and Atrogin-1, and modulating FoxO3 signaling pathways [31,32,33]. Therefore, in the present study, curcumin was selected as a positive control to benchmark the efficacy of postbiotics in the prevention of sarcopenia. Among various postbiotic candidates, *L. plantarum* beLP1 was selected because of its prevalence in kimchi and prior evidence of anti-inflammatory properties [34].

The aim of this study was to test whether the postbiotic strain *Lactobacillus plantarum* beLP1 isolated from kimchi effectively protects against dexamethasone-induced sarcopenia in cellular and animal sarcopenia models. We evaluated cell viability, cell diameters, muscle weight, muscle strength, and the expression of the muscle atrophy markers MuRF1 and Atrogin-1 to identify the efficacy and mechanisms of beLP1 using a heat-killed beLP1 strain. This study aimed to determine whether beLP1 influences AKT activation, which plays a crucial role in regulating muscle degradation pathways in dexamethasone-induced sarcopenia.

## 2. Materials and Methods

### 2.1. Postbiotic Lactobacillus plantarum 1 (beLP1)

The beLP1 postbiotics used in this study were provided by bereum Co., Ltd. (Wonju, Republic of Korea). They were derived from *L. plantarum* strains isolated from kimchi in 2023. For inactivation, beLP1 postbiotic was heat-killed at 110 °C for 25 min, with inactivation confirmed based on the absence of colony growth on agar plates. The concentration of the inactivated bacterial strain was 3.0 × 10^12^ cells per gram.

### 2.2. Establishment of the Cellular Sarcopenia Model and Cell Viability Assay

Experiments were conducted using the mouse-derived skeletal muscle cell line C2C12. The cells were cultured in Dulbecco’s Modified Eagle’s medium (DMEM, high glucose, Sigma-Aldrich, St. Louis, MO, USA) supplemented with 10% fetal bovine serum (Access Biologicals, LLC, Vista, CA, USA) and 1% penicillin/streptomycin (Sigma-Aldrich). The culture conditions were maintained at 37 °C in a 5% CO_2_ incubator. C2C12 myoblasts were seeded at 1.0 × 10^5^ cells/mL in 24-well plates. When the cells reached 90% confluence, the growth medium was replaced with differentiation medium containing 2% horse serum (WELGENE Inc., Daegu, Republic of Korea) and 1% penicillin/streptomycin to induce myotube differentiation. The cells were differentiated for six days, and the medium was changed every two days. On the fourth day of differentiation, beLP1 was diluted with the differentiation medium and added to the cells at 50, 100, and 250 μg/mL. After 48 h of beLP1 treatment, C2C12 myotubes were treated with 100 μM of dexamethasone (Sigma-Aldrich) to establish a C2C12 myotube damage model.

For the 3-(4,5-dimethylthiazol-2-yl)-2,5-diphenyltetrazolium bromide (MTT) assay, 50 μL of MTT solution (5 mg/mL) was added to each well, and the cells were incubated for 2 h. After removing the medium, the formazan crystals formed were dissolved in dimethyl sulfoxide. Optical density was measured at 595 nm using an FLx800 microplate reader (BioTek Instrument Inc., Winooski, VT, USA). The untreated group was used as the control group to compare cell viability.

### 2.3. Measurements of Myotube Diameters

Differentiated C2C12 myotubes were prepared with Giemsa staining to measure their diameter. Briefly, C2C12 cells were washed with cold PBS and fixed in 100% methanol at −20 °C for 5 min. After fixation, the cells were washed again with PBS and air-dried. Fixed cells were first stained with May–Grünwald solution (Sigma-Aldrich) for 5 min at 21 ± 3 °C, followed by PBS washing. Subsequently, cells were stained with Giemsa solution (Sigma-Aldrich) for 15 min at 21 ± 3 °C and rinsed with PBS. Stained myotubes were observed under an optical microscope (Nikon, Tokyo, Japan) at 200× magnification. The diameters of 100 myotubes were measured from at least 10 randomly selected fields using ImageJ 64-bit Java 1.8.0.170 software (National Institutes of Health, Bethesda, MD, USA).

### 2.4. Animal Experiments

Based on cell-based experiments, an animal study was conducted using Sprague–Dawley (SD) rats to investigate the mechanism by which beLP1 alleviates sarcopenia in vivo. Male SD rats (average weight: 180 g, 6 weeks old) were purchased from DBL Inc. (Eumseong, Republic of Korea). The animals were housed under controlled environmental conditions, including a temperature of 21 ± 3 °C, humidity of 45 ± 10%, and a 12 h light–dark cycle. They were given standard rodent chow (RodFeed; DBL Inc.) and sterilized water ad libitum. After 1 week of adaptation, the rats were randomly divided into five groups (n = 7 per group). The Normal group (Nor) was not treated with dexamethasone, whereas the control group (Con) was treated with dexamethasone only. Doses of 1 mg/kg (low-dose group: L.beLP1) and 3 mg/kg (high-dose group: H.beLP1) beLP1 were applied, and curcumin was applied at 10 mg/kg as a standard positive control. Following the adaptation period, each experimental group, except for the Nor and Cor groups, received 400 μL of either beLP1 or curcumin diluted in saline via oral gavage once daily at 12:00 p.m. for two weeks. Nor and Con received equivalent volumes of saline for two weeks. Following the pretreatment period, sarcopenia was induced by intraperitoneal injection of dexamethasone (600 μg/kg) in Con, L.beLP1, H.beLP1, and Cur over five days. Oral administration of beLP1 or curcumin was continued in parallel with dexamethasone treatment. At the end of the treatment period, the animals were sacrificed via CO_2_ inhalation. After sacrifice, the weights of the gastrocnemius, tibialis anterior, soleus, and plantaris muscles were measured for each group. All animal experiments were approved by the Yonsei University Institutional Animal Care and Use Committee (YWCI-202409-009-01).

### 2.5. Grip Strength Test

To evaluate the muscle function-preserving effects of beLP1, grip strength was measured using a grip strength meter (Jeongdo Bio & Plant Co., Seoul, Republic of Korea). During the measurement, each rat was gently held by the tail and allowed to grasp the meter while being pulled backward slowly to record grip strength. The grip strength test was conducted three times: before drug administration, after drug administration, and before the end of the experiment. Each rat underwent three measurements per test session.

### 2.6. Measurement of Muscle Volume Using Micro-Computed Tomography

On the sixth day, after five consecutive days of intraperitoneal dexamethasone injection, micro-computed tomography (micro-CT) was performed to assess the preventive effect of beLP1 against sarcopenia by comparing muscle volumes before sacrifice. Imaging was conducted using a SkyScan 1076 micro-CT system (Bruker, Bermen, Germany) with a resolution of 30 μm. The scanning parameters were configured as follows: 100 kV, 100 mA, 790 ms exposure time, and a rotational step of 1.2° per scan. The animals were maintained under anesthesia during the imaging process. To enhance the image quality, flat-field correction was applied before scanning, and beam-hardening correction was implemented during the reconstruction process. Muscle volume was quantified for the gastrocnemius and tibialis anterior using CT Analyzer 1.11. (Bruker, Germany) to generate 2D models of the scanned muscle structures.

### 2.7. Western Blotting Analysis

For the in vitro study, differentiated C2C12 myotubes were pretreated with beLP1 for 48 h and subsequently exposed to 100 μM of dexamethasone for 24 h to induce a cellular model of sarcopenia. After treatment, cells cultured in 6-well plates were lysed using PRO-PREP™ Protein Extraction Solution (iNtRON Biotechnology Inc., Seongnam, Republic of Korea). For the in vivo study, gastrocnemius muscle tissues were collected from each experimental group, rapidly frozen in liquid nitrogen, and stored for further analysis. Before protein extraction, the tissue was homogenized using PRO-PREP™ Protein Extraction Solution. The homogenized samples were centrifuged to remove tissue debris, and the supernatants were collected. The protein concentration was determined using the Bradford assay (Bio-Rad, Hercules, CA, USA). Western blotting was conducted following the same procedure as described above. The primary antibodies used in this study were Atrogin-1 (ab168372, Abcam, Cambridge, UK), MuRF1 (SC-32920, Santa Cruz Biotechnology, Santa Cruz, CA, USA), FoxO3α (#2497, Cell Signaling Technology, Danvers, MA, USA), p-AKT (Ser473) (#9271, Cell Signaling Technology), AKT (#9272, Cell Signaling Technology), and β-actin (#4970, Cell Signaling Technology). All primary antibodies were diluted at 1:2500 before use. Following primary antibody incubation, the membrane was washed three times with TBS-T and incubated at room temperature (20~22 °C) for 1 h with horseradish peroxidase (HRP)-conjugated secondary antibody (HRP-linked anti-rabbit antibody, #7074, Cell Signaling Technology) diluted at 1:5000. After the membranes were washed three more times with TBS-T, protein expression was visualized using WestGlow™PICO PLUS Chemiluminescent Substrate (Biomax Inc., Guri, Republic of Korea). Image analysis was performed using a ChemiDoc™ Imaging System (Bio-Rad), and protein expression was quantified using ImageJ 64-bit Java 1.8.0.170 software (National Institutes of Health, Frederick, MD, USA). The expression levels of the target proteins were normalized to those of β-actin.

### 2.8. Statistical Analysis

One-way ANOVA with Dunnett’s post hoc test was used to analyze data in multiple groups whereas *t*-tests were applied to data in two groups. Sample sizes of n = 7 and n = 3 were determined to achieve 80% power to detect a 20% difference in muscle weight and protein expression, respectively. Data are expressed as mean ± standard error of the mean. Statistical significance was set at *p* < 0.05, 0.01, and 0.001.

## 3. Results

### 3.1. beLP1 Prevents Dexamethasone-Induced Cell Damage in C2C12 Myotubes

**Figure 1:**
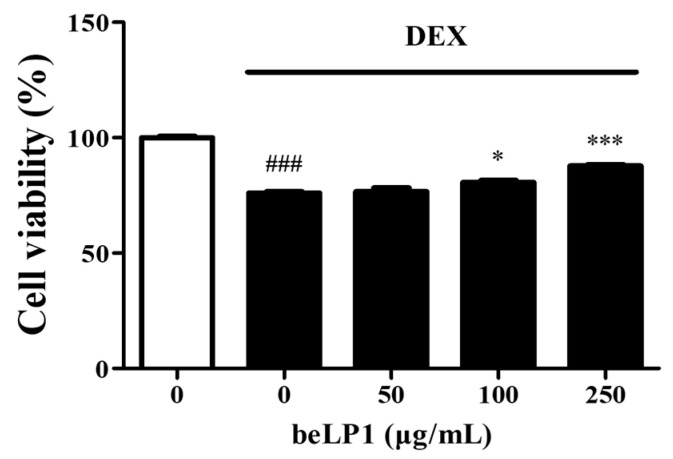
Effects of beLP1 on dexamethasone-induced cell damage in C2C12 myotubes. C2C12 myoblasts are cultured and differentiated for four days, with the differentiation medium replaced every two days. After differentiation, the cells are treated for 48 h with beLP1 diluted in the differentiation medium at concentrations of 0, 50, 100, and 250 μg/mL. Subsequently, the cells are incubated with 100 μM of dexamethasone for an additional 24 h to induce damage. Data are expressed as the mean ± standard error of the mean (SEM) (n = 4). ^###^ *p* < 0.001 compared with the untreated group. * *p* < 0.05 and *** *p* < 0.001 compared with the dexamethasone-induced cell damage group.

To evaluate the effect of beLP1 on dexamethasone-induced damage in C2C12 myotubes, cell viability was assessed using the MTT assay. The results showed that cell viability in the cells treated with dexamethasone alone decreased to 76.10% ± 0.99%. However, in the groups treated with dexamethasone and beLP1, cell viability significantly increased in a dose-dependent manner, with values of 76.70% ± 2.72% (50 μg/mL), 80.71% ± 1.31% (100 μg/mL), and 87.76% ± 1.15% (250 μg/mL) (Figure 1).

### 3.2. beLP1 Increases Myotube Diameter Reduced by Dexamethasone in C2C12 Myotubes

**Figure 2:**
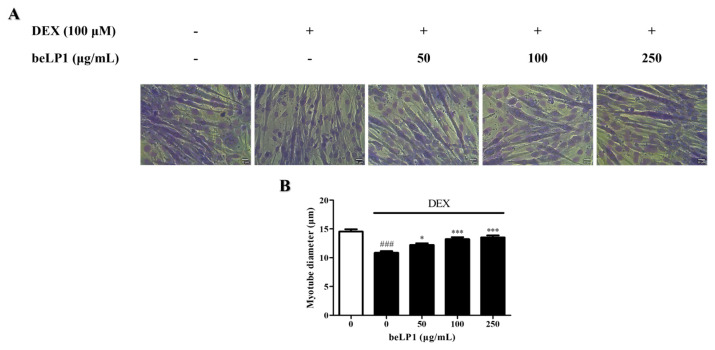
Effects of beLP1 on myotube diameters in dexamethasone-induced C2C12 myotube atrophy model: (**A**) Representative images of Giemsa staining (scale bar: 20 μm). (**B**) Quantification of myotube diameters from Giemsa-stained images. Data are expressed as the mean ± standard error of the mean (SEM). ^###^ *p* < 0.001 compared with the untreated group. * *p* < 0.05 and *** *p* < 0.001 compared with the dexamethasone-induced C2C12 myotube atrophy group.

The effect of beLP1 on myotube diameters was assessed via Giemsa staining of the C2C12 myotubes. Dexamethasone treatment decreased myotube diameter, indicating muscle atrophy. However, beLP1 treatment significantly increased the diameter of atrophic myotubes. The diameter was similar to that of the untreated group, especially at 250 µg/mL. These findings suggest that beLP1 can help prevent dexamethasone-induced sarcopenia without causing cytotoxicity (Figure 2).

### 3.3. beLP1 Decreases Atrogin-1 Protein Expression in Dexamethasone-Induced C2C12 Myotube Atrophy Model

**Figure 3:**
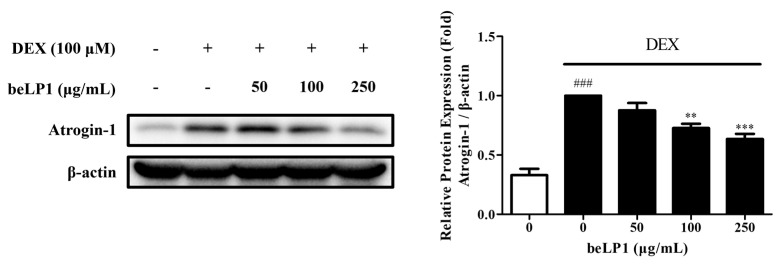
Effects of beLP1 on Atrogin-1 protein expression in the dexamethasone-induced C2C12 myotube atrophy model. Cell lysates from C2C12 myotubes are collected and blotted using an Atrogin-1 antibody and then subjected to Western blot analysis. Protein expression is quantified relative to the loading control β-actin. Data are expressed as the mean ± SEM (n = 3). ^###^ *p* < 0.001 compared with the untreated group. ** *p* < 0.01 and *** *p* < 0.001 compared with the dexamethasone-induced C2C12 myotube atrophy group.

To assess the expression of Atrogin-1, which is involved in the inhibition and degradation of muscle protein synthesis, Western blot analysis was performed using C2C12 myotubes. The results showed that, compared to the untreated group, the dexamethasone-induced C2C12 myotube atrophy group exhibited a significant increase in the expression of Atrogin-1, a key muscle degradation-related protein. However, compared to the untreated group, the beLP1-treated groups (50, 100, and 250 μg/mL) showed reduced Atrogin-1 expression (Figure 3). These findings indicated that beLP1 may help suppress muscle protein degradation by downregulating Atrogin-1 expression in dexamethasone-induced C2C12 myotube atrophy model.

### 3.4. beLP1 Reduces Muscle Volume Loss in Dexamethasone-Induced Sarcopenic Rat Model

**Figure 4:**
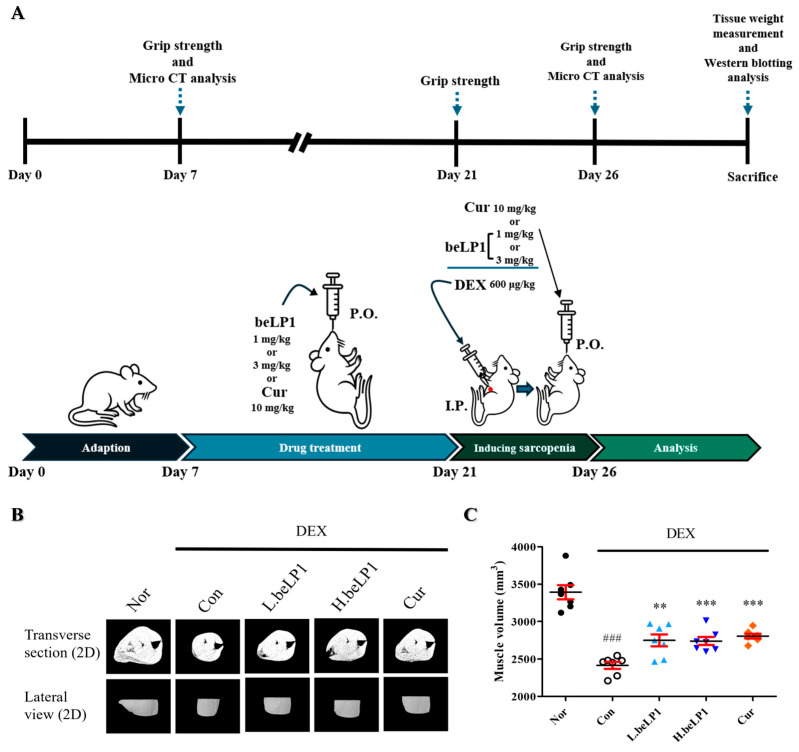
Effects of beLP1 oral administration on the muscle volume of rats with dexamethasone-induced sarcopenia: (**A**) Schematic diagram depicting the timeline of drug administration and physical experiments in vivo. (**B**) Micro-computed tomography (micro-CT). The scan results are obtained on the final day of dexamethasone administration, before sacrifice, by performing micro-CT analysis twice. (**C**) Effects of the oral administration of beLP1 on the muscle volume of dexamethasone-induced sarcopenia rats measured using micro-CT. Data are expressed as the mean ± SEM (n = 7). ^###^ *p* < 0.001 compared with Nor. ** *p* < 0.01 and *** *p* < 0.001 compared with Con.

An experimental setup was designed to evaluate the effects of beLP1 and curcumin on dexamethasone-induced sarcopenia (Figure 4A). Before sacrifice, micro-CT imaging was performed to compare muscle volumes across groups and assess the protective effect of beLP1 against sarcopenia (Figure 4B). Regions of interest were selected to include the gastrocnemius and tibialis anterior muscles of the hind limbs. Muscle volume was quantified using CT Analyzer 1.11. software, allowing for three-dimensional reconstruction and volumetric analysis. A significant reduction in muscle volume was found in the dexamethasone-treated groups compared to that in Nor. However, in the beLP1-treated groups, the decline in muscle volume was significantly alleviated, suggesting an anti-sarcopenic effect (Figure 4C).

### 3.5. beLP1 Reduces Muscle Weight Loss in Dexamethasone-Induced Sarcopenic Rat Model

**Figure 5:**
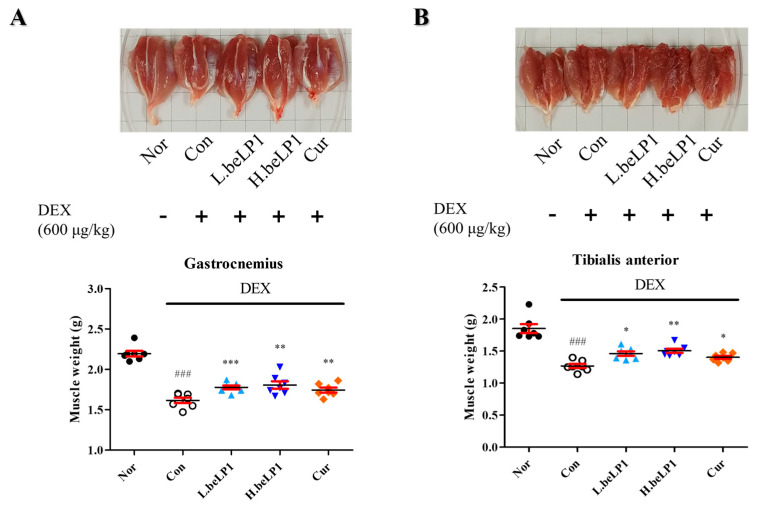

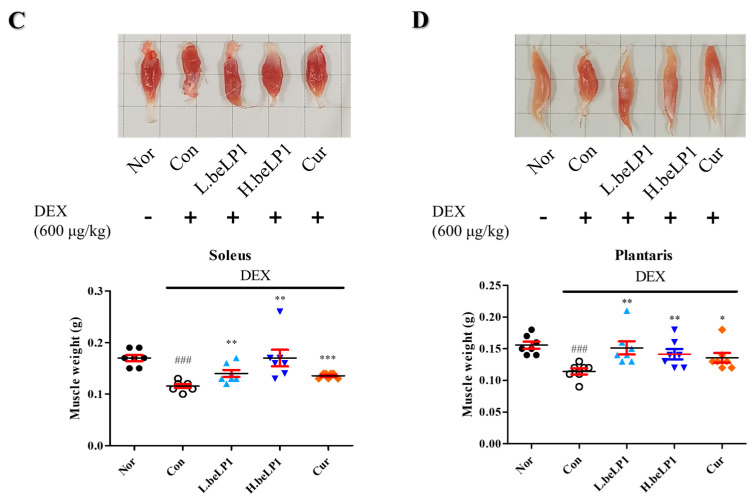
Effects of the oral administration of beLP1 on the muscle weight of dexamethasone-induced sarcopenic rats. Four types of skeletal muscles (gastrocnemius, tibialis anterior, soleus, and plantaris) were isolated from each group of Sprague–Dawley (SD) rats and weighed for analysis: (**A**) gastrocnemius, (**B**) tibialis anterior, (**C**) soleus, and (**D**) plantaris. Data are expressed as the mean ± SEM (n = 7). ^###^ *p* < 0.001 compared with Nor. * *p* < 0.05, ** *p* < 0.01, and *** *p* < 0.001 compared with the Con.

To compare muscle mass among groups, four leg muscles (gastrocnemius, tibialis anterior, soleus, and plantaris) were isolated and weighed after sacrifice. Among all muscle groups, the dexamethasone-only group (Con) significantly reduced muscle weight compared to Nor. However, in the beLP1-treated groups, the decrease in muscle weight was alleviated, indicating a protective effect against dexamethasone-induced muscle loss (Figure 5).

### 3.6. beLP1 Improves Muscle Strength in Dexamethasone-Induced Sarcopenic Rat Model

**Figure 6:**
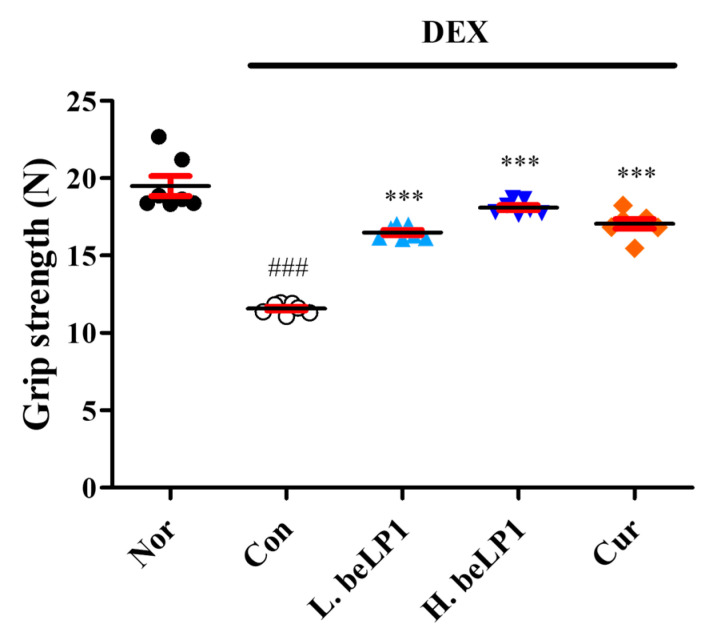
Effects of beLP1 oral administration on the muscle strength of dexamethasone-induced sarcopenic rats. The grip strength of each group is measured before sacrifice. Each result represents the average of 3 tests per animal. Data are expressed as the mean ± SEM (n = 7). ^###^ *p* < 0.001 compared with Nor. *** *p* < 0.001 compared with Con.

To evaluate the preventive effect of beLP1 against sarcopenia, muscle strength was compared among the groups using a grip strength meter. The grip strength, which indicates muscle strength, was significantly decreased in the dexamethasone-only (Con) group. However, in the beLP1- and curcumin-treated groups, grip strength significantly improved, indicating that the deterioration of the muscle function (strength) was mitigated (Figure 6).

### 3.7. beLP1 Decreases Atrogin-1 and MuRF1 Protein Expression in Dexamethasone-Induced Sarcopenic Rat Model

**Figure 7:**
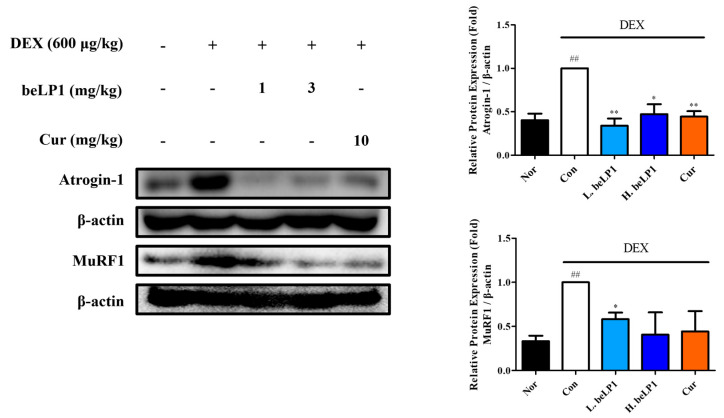
Effects of the oral administration of beLP1 on Atrogin-1 and MuRF1 protein expression in dexamethasone-induced sarcopenic rats. Tissue lysates from homogenized gastrocnemius of SD rats are collected and blotted using specific antibodies, including Atrogin-1 and MuRF1, and then subjected to Western blot analysis as described in Section 2.7. Protein expression is quantified relative to the loading control β-actin. Data are expressed as the mean ± SEM (n = 3 per group). ^##^ *p* < 0.01 compared with Nor. * *p* < 0.05 and ** *p* < 0.01 compared with Con.

To assess the levels of Atrogin-1 and MuRF1 expression, which are involved in the inhibition and degradation of muscle protein synthesis, Western blot analysis was performed using gastrocnemius muscle tissue extracted from each group. The results showed that, compared to Nor, the dexamethasone-only group (Con) exhibited a significant increase in the expression of Atrogin-1, a key muscle degradation-related protein. However, compared to Con, the beLP1-treated groups (1 and 3 mg/kg) and curcumin-treated group (10 mg/kg) showed reduced Atrogin-1 expression. Additionally, a significant reduction in MuRF1 expression was observed in L.beLP1 compared to that in Con (Figure 7). These findings indicated that beLP1 may help suppress muscle protein degradation by downregulating Atrogin-1 and MuRF1 expression in dexamethasone-induced sarcopenia.

### 3.8. beLP1 Decreases FoxO3α Protein Expression and Increases AKT Phosphorylation in Dexamethasone-Induced Sarcopenic Rat Model

**Figure 8:**
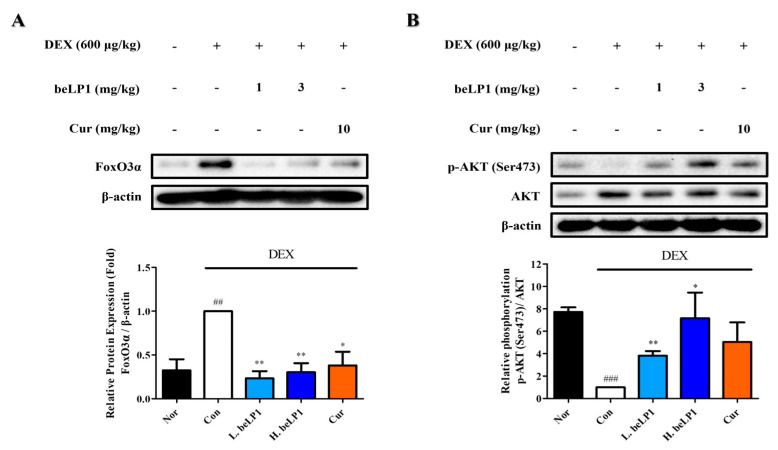
Effects of the oral administration of beLP1 on Forkhead box O3α (FoxO3α) protein expression and AKT phosphorylation in rats with dexamethasone-induced sarcopenia: (**A**) Effect of beLP1 on FoxO3α protein expression in a dexamethasone-induced sarcopenia rat model. Tissue lysates from the homogenized gastrocnemius of SD rats are collected and blotted using specific antibodies, including FoxO3α, and then subjected to Western blot analysis as described in Section 2.7. Protein expression is quantified relative to the loading control β-actin. Data are expressed as the mean ± SEM (n = 4 per group). ^##^ *p* < 0.01 compared with Nor. * *p* < 0.05 and ** *p* < 0.01 compared with Con. (**B**) Effect of beLP1 on the phosphorylation of AKT serine proteins in a dexamethasone-induced sarcopenia rat model. Tissue lysates from the homogenized gastrocnemius of SD rats are collected and blotted using specific antibodies, including p-AKT (Ser473), and then subjected to Western blot analysis as described in Section 2.7. Phosphorylation levels are quantified relative to the loading control AKT. Data are expressed as the mean ± SEM (n = 4 per group). ^###^ *p* < 0.001 compared with Nor. * *p* < 0.05 and ** *p* < 0.01 compared with Con.

The expression of FoxO3α, a transcription factor that regulates the expression of Atrogin-1 and MuRF1, was examined using gastrocnemius muscle tissue extracted from each group. Western blot analysis performed to assess the effect of beLP1 on FoxO3α expression revealed that the oral administration of beLP1 (1 and 3 mg/kg) or curcumin (10 mg/kg) significantly reduced FoxO3α expression compared to dexamethasone alone (Figure 8A).

The phosphorylation level of AKT (Ser473), a protein involved in cell survival, apoptosis regulation, and cell proliferation, as well as a key regulator of FoxO3α phosphorylation, was examined in the gastrocnemius muscle tissue extracted from each group. Western blotting was performed to assess the effect of beLP1 on AKT (Ser473) phosphorylation. The experimental results showed that the phosphorylation level of AKT was significantly lower in the dexamethasone-only group (Con) than in the normal group (Nor). However, compared to dexamethasone, beLP1 (1 and 3 mg/kg) and curcumin (10 mg/kg) tended to increase AKT phosphorylation. The group treated with beLP1 (1 mg/kg) showed a significant increase in AKT phosphorylation (Figure 8B). These findings suggest that beLP1 attenuates muscle protein degradation by inducing AKT phosphorylation in dexamethasone-induced sarcopenia.

## 4. Discussion

Sarcopenia is a disease characterized by the loss of muscle mass and muscle strength and aging. It significantly impacts the function and activities of older adults and is a serious medical condition [4]. The pathophysiology of sarcopenia is complicated and involves the activation of pathways involved in muscle protein degradation, which is a major reason for its development [35]. Among the various pathways, the UPS is the key system responsible for muscle protein degradation, which is mainly regulated by MuRF1 and Atrogin-1 [11]. In the current study, we evaluated the potential of *L. plantarum* beLP1 as a functional ingredient in regulating these protein degradation pathways.

Our findings indicated that beLP1 improves dexamethasone-induced sarcopenia in animal models, as evidenced by increased muscle mass and the decreased expression of sarcopenia markers. These results are consistent with previous studies [27] demonstrating the beneficial effects of postbiotics on muscle health. Cell-based experiments reinforced the non-toxicity of beLP1 in C2C12 myotubes. Despite dexamethasone treatment, the cell viability increased upon beLP1 treatment, showing a protective effect (Figure 1), indicating that dexamethasone-induced cellular damage can be prevented through beLP1. The observed ~24% decrease in cell viability following dexamethasone treatment represents an early cellular event such as apoptosis, which is an upstream trigger of muscle protein degradation. Apoptotic signaling pathways activate E3 ubiquitin ligases including MuRF1 and Atrogin-1 via FoxO3α, leading to the proteolysis of muscle proteins and subsequent sarcopenia [14,36]. Although in vitro viability loss does not directly equate to measurable sarcopenia phenotypes such as decreased grip strength or muscle mass, it provides mechanistic insights into the early degenerative processes. Thus, the effects of beLP1 to increase cell viability suggest a protective role in preventing the initiation of these muscle-degrading pathways. In addition to preserving cell viability, beLP1 also alleviated dexamethasone-induced morphological changes in C2C12 myotubes. Specifically, beLP1 significantly increased the myotube diameter that had been reduced by dexamethasone, and, at the highest concentration, the diameter was comparable to that of the untreated group (Figure 2).

The protective effects of beLP1 on the muscles have been verified in animal models. Micro-CT analysis showed that the muscle volume was reduced in the group treated with dexamethasone alone. However, this reduction was less pronounced in the group that received beLP1 in combination with dexamethasone (Figure 4). When the weights of individual muscles (gastrocnemius, tibialis anterior, soleus, and plantaris) were measured, a significant decrease was observed in the dexamethasone-only group. In the beLP1-treated groups, the muscle weight was significantly higher, despite dexamethasone treatment (Figure 5). Additionally, in the grip strength test, the beLP1 groups showed improved muscle strength compared to the dexamethasone-only group (Figure 6).

To examine these molecular pathways in greater detail, we quantified the expression of muscle breakdown regulators. Western blot analysis revealed the upregulation of Atrogin-1 and MuRF1 in the group treated with dexamethasone and the downregulation of these proteins in the beLP1-treated group (Figure 3 and Figure 7). These data indicate that the UPS activation might be inhibited by beLP1 and that muscle protein degradation is suppressed. Further, the transcription factor FoxO3α, which is responsible for the upregulation of muscle protein degradation via MuRF1 and Atrogin-1, was downregulated in the beLP1-treated group (Figure 8A). Considering that FoxO3α is the upstream regulator of the pathways involved in skeletal muscle degradation, the findings indicate that beLP1 may inhibit muscle degradation by regulating the FoxO3α signaling pathway.

The AKT signaling pathway is a major regulator of muscle protein synthesis and degradation [37]. AKT inhibits FoxO3α activity by phosphorylating FoxO3α, stimulating muscle protein synthesis, and preventing degradation [38]. We examined the role of beLP1 in AKT (Ser473) phosphorylation (Figure 8B) and observed that the AKT pathway can be activated by beLP1 to prevent muscle protein breakdown. Further studies are needed to elucidate the exact mechanism by which beLP1 affects the AKT signaling pathway in muscle protection.

The dosages of beLP1 used in this study (1 and 3 mg/kg) were selected based on previous postbiotic studies [39,40], which applied approximately 3 billion cell/kg body weight in rodent models. Given that beLP1 contains 3.0 × 10^12^ cell/g, these doses (1 and 3 mg/kg) correspond to biologically relevant cell doses. Similarly, the curcumin dose (10 mg/kg) was chosen in accordance with the prior literature [41].

The efficacy of beLP1 was also compared to curcumin, which has been widely studied as a natural compound with anti-sarcopenic effects.

Curcumin has been reported to alleviate glucocorticoid-induced muscle wasting through its anti-inflammatory and anti-atrophic activities. In line with previous reports, our study confirmed that curcumin mitigated dexamethasone-induced reductions in muscle mass and strength [31,32,33]. Notably, beLP1 exhibited comparable or even greater effects in some parameters, suggesting its potential as an alternative to existing natural precautions.

These findings indicate that postbiotics are novel functional additives for the prevention of sarcopenia. Probiotics exhibit gut microbiota- and immune-modulating activities, but since they use living bacteria, their use in immunocompromised older adults is associated with infection risk [24]. However, postbiotics, derived from microbial metabolites and cellular components, are safer and exhibit the same health-promoting physiological effects as probiotics. In this study, we demonstrated that *L. plantarum* beLP1 holds potential as a functional component in targeted sarcopenia precaution by confirming its effectiveness in inhibiting the muscle protein degradation pathway.

Previous studies have reported the beneficial effects of various postbiotics, such as *Lactobacillus gasseri* CP2305, which improved stress-related symptoms and gut health, and *Lactobacillus rhamnosus* GG-derived components, which showed immunomodulatory properties [42,43]. Compared to these strains, beLP1 derived from *L. plantarum* exhibited a distinct muscle-protective effect in glucocorticoid-induced sarcopenia, suggesting strain-specific postbiotic functionality. This highlights the importance of selecting specific postbiotic strains based on targeted therapeutic applications.

In addition to direct effects on muscle, postbiotics such as beLP1 may exert systemic effects by modulating gut microbiota composition and host immune responses. Given that beLP1 is derived from *L. plantarum*, a strain known to influence intestinal homeostasis and inflammatory signaling, its systemic impact warrants further investigation. These effects may be especially relevant for immunocompromised populations, in which live probiotics pose infection risks, whereas postbiotics offer a safer alternative with preserved bioactivity [25].

Despite our promising results, this study had several limitations. First, although we observed the effects of beLP1, the specific bioactive compounds responsible for these effects have not been identified. Further studies are needed to isolate and characterize the active components within beLP1 to better understand its mechanism of action. Second, although molecular markers of muscle degradation were analyzed, we did not conduct histological assessments such as hematoxylin and eosin staining or immunohistochemistry. Such studies should assess structural changes in muscle fibers, thereby supporting the effects of beLP1 against sarcopenia.

## 5. Conclusions

**Figure 9:**
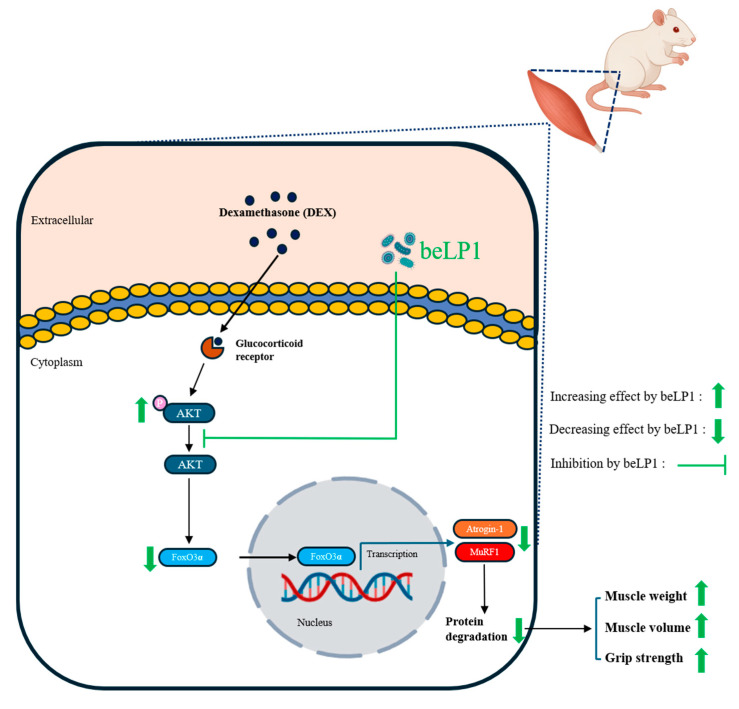
Schematic diagram of anti-sarcopenic activity of postbiotic beLP1 on dexamethasone-induced sarcopenic rat model.

This study demonstrated that beLP1 reduces muscle degradation and preserves strength in a rat model of sarcopenia, suggesting potential as a preventive agent (Figure 9). However, clinical trials are needed to confirm efficacy and safety in humans.

## Data Availability

The data supporting the findings of this study are available from the corresponding author upon request.

## Abbreviations

The following abbreviations are used in this manuscript:

Nor	Normal group
Con	Control group
Cur	Curcumin-treated group
L.beLP1	Low-dose beLP1-treated group
H.beLP1	High-dose beLP1-treated group
MTT	3-(4,5-dimethylthiazol-2-yl)-2,5-diphenyltetrazolium bromide
SD	Sprague–Dawley
Micro-CT	Micro-computed tomography
UPS	Ubiquitin–proteasome system
